# Self-Reinforced Nylon 6 Composite for Smart Vibration Damping

**DOI:** 10.3390/polym13081235

**Published:** 2021-04-11

**Authors:** Bidita Salahuddin, Rahim Mutlu, Tajwar A. Baigh, Mohammed N. Alghamdi, Shazed Aziz

**Affiliations:** 1 ARC Centre of Excellence for Electromaterials Science and Intelligent Polymer Research Institute, University of Wollongong, Innovation Campus, Squires Way, North Wollongong, NSW 2522, Australia; 2 School of Mechanical, Materials, Mechatronic and Biomedical Engineering, University of Wollongong, Wollongong, NSW 2522, Australia; 3 Department of Mechanical and Production Engineering, Islamic University of Technology, Gazipur 1704, Bangladesh; tajwar.azim@gmail.com; 4 Department of Mechanical Engineering Technology, Yanbu Industrial College, Yanbu Al-Sinaiyah City 41912, Saudi Arabia; alghamdim@rcyci.edu.sa; 5 School of Chemical Engineering, The University of Queensland, Brisbane, QLD 4072, Australia

**Keywords:** self-reinforced composites, nylon 6, polycaprolactam, polymer coil actuator, 3D extrusion printing, vibration damping, polymer crystallinity

## Abstract

Passive vibration control using polymer composites has been extensively investigated by the engineering community. In this paper, a new kind of vibration dampening polymer composite was developed where oriented nylon 6 fibres were used as the reinforcement, and 3D printed unoriented nylon 6 was used as the matrix material. The shape of the reinforcing fibres was modified to a coiled structure which transformed the fibres into a smart thermoresponsive actuator. This novel self-reinforced composite was of high mechanical robustness and its efficacy was demonstrated as an active dampening system for oscillatory vibration of a heated vibrating system. The blocking force generated within the reinforcing coiled actuator was responsible for dissipating vibration energy and increase the magnitude of the damping factor compared to samples made of non-reinforced nylon 6. Further study shows that the appropriate annealing of coiled actuators provides an enhanced dampening capability to the composite structure. The extent of crystallinity of the reinforcing actuators is found to directly influence the vibration dampening capacity.

## 1. Introduction

Vibrations in structural assembly reduce structural stability, position precision, flexibility, and noise control. When a mechanical or material system encounters high-frequency vibration, a substantial quantity of passive heat can be produced due to the viscoelastic losses in the system [1]. Active heating is also a major issue when vibration is produced within a complex mechanical system. This is a concerning issue for large structures such as aircraft or automobiles, as well as small assemblies like electronics. Polymers, when engineered appropriately, are excellent materials for damping passive vibration [2]. For instance, the unique properties of viscoelastic polymers include their enormous flexibility and excellent energy dissipation capability, though the elastic modulus and damping capacity of such polymers are very delicate when the vibration frequency is changed (i.e., the frequency of loading) and temperature [3]. A vibration damper fabricated from such viscoelastic polymers can be formed either as an insert to place in a cavity within the structure or a laminate that is positioned between two surfaces of the structure [4,5,6]. However, a self-heating effect due to mechanical energy dissipation within a polymeric damper is also a critical problem when the damper experiences prolonged cyclic loading or excessive vibration [7,8]. This self-heating effect typically originates from the internal friction of the polymer molecules, which leads to hysteretic behaviour [9]. The heat resulting from the dissipative processes triggers the temperature increase of the loaded structure causing the self-heating effect.

In the case of vibration damping by composite materials, the presence of fillers or reinforcement creates complex internal structures in the overall composite system. The damping behaviours depend on the properties of the material, as well as on the volume or weight fraction of fillers, the interfacial phenomena, filler/reinforcement spread, and dispersion, loading direction, and plasticisation of the matrix material. Several energy dissipation mechanisms exist in fibre reinforced composites such as viscoelasticity of matrix and fibre materials, the friction generated in the fibre/matrix interfacial region, energy dissipation at cracks and interfacial delamination produced at damaged locations, viscoplasticity, and thermoelastic damping [10,11]. It has been shown that the self-reinforced composites (similar fibre and matrix materials) can withstand unwanted impact or vibrational force with less vulnerability to fibre/matrix delamination or cracks [12]. Coupling such a system response and behaviour is a challenging task but an important subject for any smart system design.

In the context of composite fabrication, 3D printing offers many advantages including high precision, cost-effectiveness, and customised geometry [13]. This offers the ability to change the mechanical properties of engineering structures, such as stiffness which can be altered with internal fabrication patterns of the engineering structure [14]. Thermoplastic polymers are the most used precursor materials for the extrusion-based fused filament 3D fabrication process, due to their inexpensiveness and suitable melting temperatures [15]. Several popular choices of thermoplastics exist such as acrylonitrile butadiene styrene, polylactic acid, polycarbonate, polyether ether ketone, and nylon [16,17]. Nonetheless, the printed materials often lack the mechanical properties to fabricate functional engineering components, which has limited the broader acceptance of this technology in practical applications [18]. To overcome this problem, reinforcements, such as fibres, are incorporated with the polymer matrix while printing, to manufacture a composite structure with improved strength and stiffness. Moreover, the effect of mechanical vibration on the crack generation and propagation of structural composites has been overlooked in terms of introducing an adaptive technology. Self-reinforcing 3D printed composites can diminish the risk of crack generation with high interfacial shear strength.

Universally, several techniques can damp mechanical vibrations, for example, by using external dampers that are highly effective for heavy assemblies. For lightweight materials and their assemblies, vibration reduction using an external damper is challenging to achieve. Exploring new materials or systems with combined characteristics of high strength, lightweight, inexpensiveness, and controlled damping capacity has now become crucial. Actuators such as piezoelectric polymers have also been widely researched for controlled vibration damping and have been found useful for the spacecraft near-DC bias motion. However, real-time control of the vibration requires voltage applied from the external power supply with feedback from an external sensing unit such as a displacement sensor. A magnetorheological elastomer-based vibration damper has been demonstrated as a smart system to synchronise itself with the time-dependent harmonic oscillatory force to reduce the vibration of the steady-state system [19]. However, it requires an external magnetic field to apply. No active use of the self-heat produced due to the mechanical vibration has been demonstrated, which can be a practical solution to trigger an on-demand vibration damping system without requiring an external facility. Researchers have manipulated the vibration frequencies of epoxy composite beams by reinforcing them with stimuli-responsive shape memory alloy fibre actuators. When electrically heated, the fibres have generated a blocking force due to their restrained condition by the constraints of the matrix. However, this blocked force has led to a rise in the free vibration frequency of the full composite shaft. Shape memory elastomeric fibres have attracted significant interest for use as vibration dampers in lightweight engineering composite structures. However, these polymers exhibit a lack of control and environmental adaptation issues in providing effective vibration resistance. Recently, highly oriented polyamide fibres were shown to be capable of acting as thermo-responsive contractile actuators while structurally reformed to twist-induced coils/springs [20]. Scalable and controlled actuation of coiled actuators can be obtained by following the specific fabrication parameters [21,22]. They also exhibit many other inherent practical advantages such as handleability, durability, and high robustness, which make them attractive reinforcement for structural devices. Therefore, these coiled actuators reinforced elastomeric composite can be used as the reinforcing filler to damp active vibration during the heat transmission process.

In a previous study, researchers have reported the damping behaviour of coiled polyamide fibre by applying a step load without actively inducing the sample [23]. However, there has been no research done that uses stimuli-responsive polymer fibres for the on-demand triggering of mechanical vibration damping using the thermal energy produced or delivered within a vibrating system. This research paper utilises commercially available highly oriented polycaprolactam (commercially known as nylon 6) fibres as the twisted/coiled actuators for mechanical vibration damping in a heated mechanical assembly. The coiled structure of the fibre will be restrained by annealing the coiled sample by heating at a temperature over the glass transition temperature(T_g_) [24] and then reinforcing it within a continuous nylon 6 matrix. The novelty of such composite lies in the integration and operational domain of the embedded thermoresponsive actuators that actively participate in absorbing system heat (heat sink), making the overall system a smart vibration damper. Coiled actuators will mostly be responsible for vibration damping due to the generation of thermoresponsive blocking force, and the unoriented thermoplastic matrix will act as the solid platform. A set of self-reinforced composites was prepared by using the nylon 6 coils heat set at different temperature. Heat setting determines the extent of crystallinity and stiffness of the coiled actuators, and they eventually manipulate the magnitude of damping factor obtained from the composites. The samples were characterised via the impact produced free vibration of a heated plastic bar. The resulting vibration response is continuously observed using a LASER displacement analyser focused on the tip of the heated plastic bar. The data is then processed and analysed based on the logarithmic decrement method to evaluate the damping ratio [25]. Based on the concept developed, it is expected that the developed self-reinforced composites can damp the vibration more effectively than that obtained from a control pure polymer damper. The current work mostly deals with the development of a novel smart composite system for adaptive vibration damping in heated assemblies. However, with further optimisation and improvements, this kind of damper can be used for real-world applications such as bushings for vibrating boilers, HVAC systems, as well as small assemblies like electronics. Coupled with motion sensors, it can also be used for ultra-stable and smart response systems. Potential future work may also include an investigation using high thermally conductive fillers such as CNT with Nylon 6 dampers for heat sink and/or self-heating system applications.

## 2. Materials and Methods

### 2.1. Materials

Commercially sourced nylon 6 fibre (Sport Fisher monofilament, 300 µm diameter, Brisbane, Australia) was used for coiled fibre fabrication. KODAK nylon 6 filament (2.85 mm diameter) was used for 3D extrusion printing. A square Teflon mould was used as the platform for the 3D printed material. Conductive silver paint (Jaycar Electronics, Brisbane, Australia) was sourced locally having an electrical conductivity of 0.02–0.1 Ω/cm^2^.

### 2.2. Fabrication of Coiled Fibres

**Figure 1:**
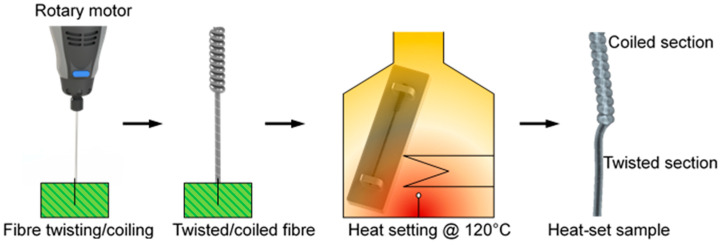
Fabrication method of a coiled fibre.

Figure 1 shows a schematic illustration of fabricating a twisted/coiled fibre used as composite reinforcement. The fibre was suspended from a motor shaft at its upper end and supported by a fixed mass hanging on the bottom end applying 10 MPa axial stress to the fibre. The bottom mass was tied opposing to the motor rotation, and each turn from the motor formed one turn in the fibre. The coiled section of twisted/coiled fibre was then placed within a convection oven and heat set above the glass transition temperature (T_g_) of nylon 6 (approx. 47 °C) [26,27], for 60 min, while both ends were still clamped to prevent any deformation. Several heat set temperatures of 50, 100, 150, and 200 °C were used to fabricate a set of four coiled samples. The fibre was then taken out from the oven and relaxed at room temperature for 2 h while still clamped. Heat setting at a temperature over T_g_ benefits the newly formed coiled shape to be permanently set. Different elevated heat setting temperature was chosen as time-based creep and stress relaxation effects are less for high-temperature annealed twisted/coiled polymer fibres [28]. Heat setting at high temperature could also have a significant effect on the polymer crystallinity, hence vibration damping, which is one of the major aims of this study.

Morphological and physical properties of the coiled section of the fibre were then performed. Scanning electron microscopy (SEM) was used to evaluate the uniformity of coils and their geometrical structure. Differential scanning calorimetry (DSC) test was conducted both as-received and coiled fibres to evaluate their melting points and confirm the sustainability of semicrystalline structure after fibre coiling. Thermogravimetric analysis (TGA) was performed to determine the drying temperature of the coiled fibres before use as composite reinforcement.

### 2.3. Fabrication of Self-Reinforced Composites

**Figure 2:**
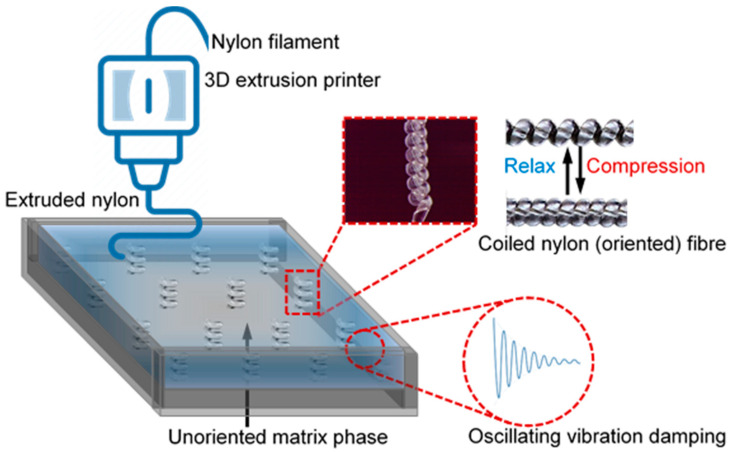
3D printing of self-reinforced composite matrix with embedded coiled fibre reinforcement as depicted in a Teflon mould (image for illustration only—not to scale and quantify).

3D printing was performed in an open-source fuse filament extrusion 3Dprinter with a heated printing stage, which helps control the covering material temperature during printing. Both the printing filament and coiled fibres were vacuum dried for 3 h at 125 °C to remove the moisture before fabrications of the composite. The printing nozzle was 400 µm in diameter and the nozzle temperature during extrusion was set to 250 °C. A Teflon mould (100 mm × 100 mm × 10 mm) was used as the printing stage where 16 coiled fibres (each 10 mm long) were vertically placed in a horizontal pattern using the melt-adhesion technique as depicted in Figure 2. The 3D printer was programmed to follow the ‘line’ infill pattern while fabricating the inside matrix structure. When deposited on the Teflon mould, the temperature of the nylon 6 matrix was ensured to be just enough for material flow, such that the coiled fibres were not affected by the molten nylon 6 matrix. This was possible due to a slightly higher melting point of the oriented coiled fibres compared to that of printed nylon 6 matrix. Once the printed material was cured, the piece of the composite was removed from the Teflon mould and polished using fine-grade sandpaper. Composite samples with 50, 100, 150, and 200 °C heat set coiled fibres were produced and named Composite 1, Composite 2, Composite 3, and Composite 4, respectively. Composite samples will be referred to in the rest of this manuscript unless indicated otherwise.

The prepared composite samples were ready for a set of characterisations upon sandpaper-polishing to reduce fatigue effects of surface cracks. The sample was then cut into rectangular pieces (100 mm × 30 mm × 10 mm) for tensile and (30 mm × 30 mm × 10 mm) for vibration damping tests. 3D printed nylon 6 only samples were also fabricated without using any coiled fibres using the process explained above, and these were used as control samples. Evaluation of tensile properties gives an estimation of interfacial adhesion in between the coiled fibre and matrix materials, which is crucial for the ultimate tensile properties of the composites. Although the orientation of the filler fibres could also play a significant role in the tensile properties of the composites, due to the scope of the current work, this factor was not considered for investigation.

### 2.4. Vibration Damping Test 

The vibration damping measurement setup consists of a conductive silver-coated polypropylene (PP) beam (100 mm × 3 mm × 1 mm), two adjacently placed as-prepared composite bars (30 mm × 30 mm × 1 mm) clamping the plastic beam at the fixed end, and LASER displacement sensor (SICK OD2-P250W150I0, SICK Pty Ltd, Melbourne, Australia) focused on the tip of the cantilever polypropylene beam. The plastic beam was Joule heated to 50 °C during the vibration tests to mimic a random temperature that can produce within a vibrating mechanical system. The free, untethered end of the plastic beam was subjected to a transverse direction impact force, and due to that, oscillatory vibration waves were produced and recorded by an e-corder data recorder unit (ED 410, e-DAQ Pty Ltd, Sydney, Australia). Calculating the damping factor of the system, including the PP beam and the novel self-reinforced composite padding, would be sophisticated. However, we were able to conduct a comparative analysis with a simplified dynamic system model using logarithmic decrement to calculate the damping factor of the effective system. The damping ratio of the harmonic vibration of the PP beam was calculated by considering upper displacement limits of 20 successive peaks that obtains the period, $\tau_{d}$. The damped natural frequency, $\omega_{d}$ of the system can be calculated using

(1) $$\omega_{d}=\frac{2\pi }{\tau_{d}}$$

The damping ratio, ζ from the logarithmic decrement, δ measured from the LASER displacement sensor using Equations (2) and (3).

(2) $$\delta =\frac{2\pi \zeta }{\sqrt{1-\zeta^{2}}}$$

(3) $$\delta =\frac{1}{n}\ln\left(\frac{d_{0}}{d_{n}}\right)$$

where n depicts the number of periods (i.e., 20 which is arbitrary), $d_{0}$ and $d_{n}$ are initial (the first peak corresponds to the time at zero), and 20th displacement peaks. Effective damping factor, $b_{e}$ of the system can be calculated with Equation (4).

(4) $$b_{e}=2\zeta \sqrt{M_{e}k_{e}}$$

where $M_{e}=\frac{33}{140}m$ and $k_{e}=\frac{3EI}{L^{3}}$ that m depicts the mass of the beam, E is Young’s modulus of PP, I is the second moment of area (i.e., $I=\frac{{bh}^{3}}{12}$) for the PP beam.

## 3. Results and Discussion

### 3.1. Properties of Coiled Fibres

**Figure 3:**
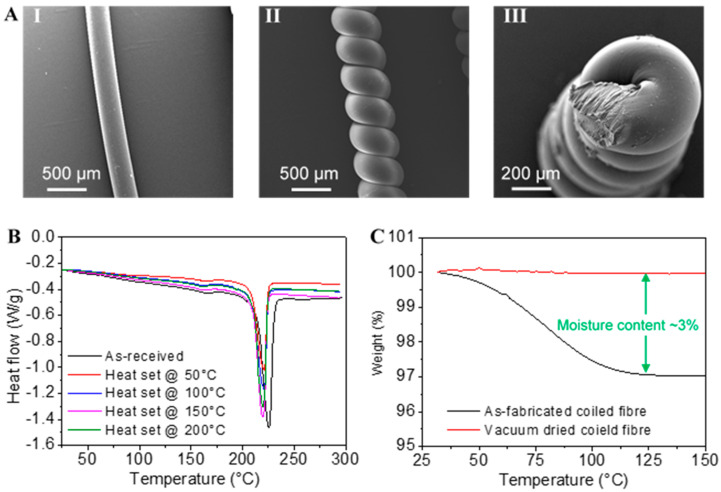
Properties of coiled fibre (actuator): (**A**) SEM images showing the coil structure, (**B**) DSC thermograms of fibres with different heat set temperature, (**C**) TGA thermograms showing the amount of moisture is non-dried and dried fibres.

Nylon 6 fibres of 300 µm diameter were used for the fabrication of coiled actuators. The microscopic morphology and shape of the actuators were investigated via SEM analysis with 10 kV accelerating potential, 35–100× magnification, and ~8 mm working distance. Figure 3A shows SEM micrographs of as-received in Figure 3A-I and coiled in Figure 3A-II nylon 6 fibres. The coiled fibre possesses some gaps between each coil, which is critical for the matrix material to strongly bond with them during composite fabrication. Figure 3A-III shows the cross-sectional view of a free-standing coiled fibre showing the persistence of coils without double-side tethering. This overtwisted coiled fibre consists of an axial hollow space that permits the melt-flown matrix material to effectively cover the helical fibre structure. The combination of mechanical twisting/coiling and heat setting ensures this new permanent shape to the fibre. The coiled fibre was then taken for DSC analysis. DSC tests (ISO 11357-1:2016) were performed on the coiled fibres from 25 to 290 °C at a heating rate of 10 °C /min in nitrogen gas (50 mL/min). Figure 3B shows the DSC curves of relevant coiled fibres, where a slight decrease of the melting points was noticed for coiled fibres compared to the precursor fibre. Such a decrease may be the result of the loss of partial crystallinity of the fibres during mechanical twisting. Table 1 shows the useful results obtained from the DSC analysis, including the deviations obtained from three samples of each type. The degree of crystallinity was estimated by considering the heat of fusion for a fully crystallised nylon 6 sample (239 J/g) [29,30]. Although the degree of crystallinity has decreased after fibre coiling, the difference is much lower with the sample heat set at 150 °C. This can be related to the classical examples of increasing crystallinity in semicrystalline polymers using an annealing technique [31,32]. The 150 °C temperature was also just enough that it had a negligible effect on material degradation, which is probably the case of having less crystallinity from a sample heat set at 200 °C. TGA (ISO 11358-1:2014) was performed to determine the drying temperature of the coiled fibres before use as composite reinforcement. Three samples of coiled fibres (heat set at 150 °C) were heated from 30 to 150 °C at a heating rate of 10 °C/min in nitrogen gas (20 mL/min). When the sample was vacuum dried for 3 h at 125 °C, there was negligible moisture left in the sample; therefore, this drying condition was used for further works.

### 3.2. Properties of Self-Reinforced Composites

**Figure 4:**
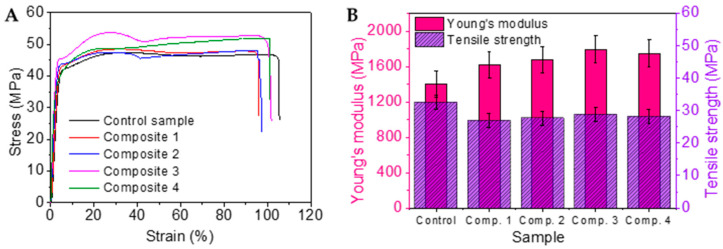
Properties of self-reinforced composites: (**A**) Tensile stress-strain curves, (**B**) Comparative tensile properties.

The 3D printed composite was mechanically tested to evaluate its tensile properties. A universal tensile tester and ISO 527-1 test method (test speed, 5 mm/min) were used to evaluate the tensile modulus and strength of the composite samples (three samples of each type). When the polymer was in the molten state during printing, all the chains were in an amorphous state. Once a line was printed, it took several minutes to decrease to the room temperature, with a similar time taken (for cooling) for other lines that were printed above the already printed lines [33,34]. This cooling happens in a very short period for most polymers to crystallize to their complete potential and to have the desired mechanical properties. Figure 4A shows the tensile stress-strain curves of the self-reinforced nylon 6 composites. Both the tensile strength at yield and ultimate tensile strength was found to be the highest for Composite 3 (also shown as Comp. 3 in Figure 4B), signifying the best fibre/matrix compatibility amongst all four composite samples. Figure 4B also shows the comparative tensile results of each composite sample together with that from a control 3D printed nylon 6 specimen. The Young’s modulus of the composite samples was relatively higher due to the contribution from highly stiff oriented nylon 6 fibres (Young’s modulus 2700 MPa) reinforcement. Composite 3 (sample with coiled fibre heat set at 150 °C) showed the highest increase of modulus owing to the excess crystallinity from the effective annealing process. 

### 3.3. Vibration Damping Test Results

**Figure 5:**
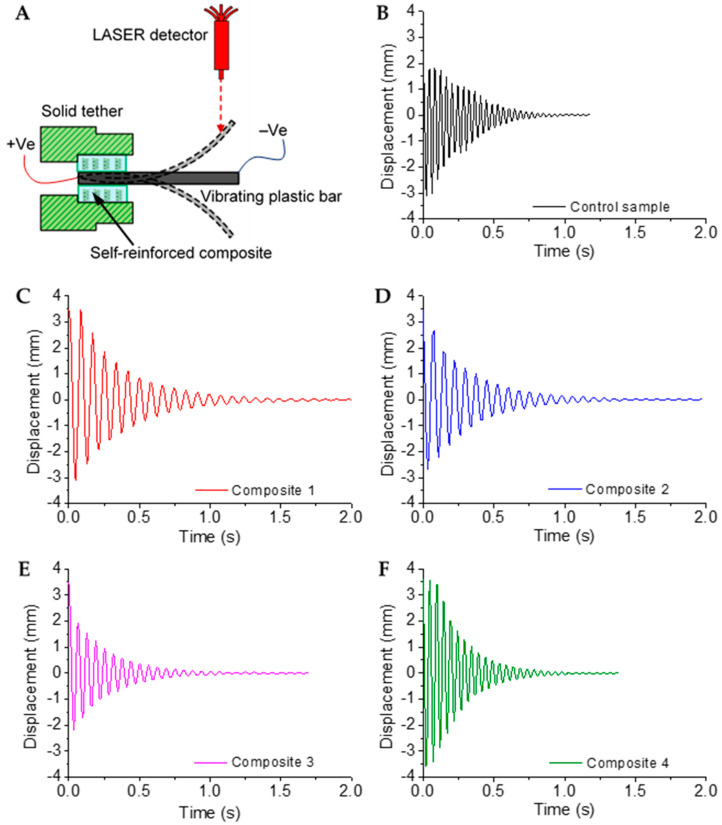
Vibration damping ability self-reinforced composites: (**A**) Schematic illustration of vibration damping measurements, (**B**) Control sample, (**C**) Composite 1, (**D**) Composite 2, (**E**) Composite 3, (**F**) Composite 4.

The damping behaviour of an impact-produced vibration on a heated plastic bar was estimated using a LASER displacement analyser. Figure 5A shows the schematic illustration of the test setup used for measuring the damp harmonic oscillatory vibration. Three samples each of the control samples and composites 1-4 were tested to ensure the reproducibility of the result. The vibrating plastic bar was subjected to resistive heating (50 °C) by applying a constant 5V DC voltage. The heat was then conducted through the adjacent composite matrices and activated the coiled actuators for force generation. The bars of the self-reinforced composites (Figure 5) helped to damp the oscillation of the plastic bar much faster than the bars of the printed control sample (Figure 5B). It should be noted that the initial displacement input in the control sample was much smaller (~1.8 mm) than the composite samples (approx. 3.5 mm). When heated and vibrated, the reinforcing coiled fibres tried to generate length contraction that originated from a fibre untwist during heating [20,35]. Due to the restrained condition of fibres by the constraints of the matrix, our coiled fibres generated a blocked force instead of length contraction. This blocking force actively repeals the mechanical energy produced by vibration and delivers faster damping. Among the composites used, Composite 3 exhibited the best performance with a vibration-damping factor of 1.31 (±0.09) compared to that of 1.08 (±0.1) obtained for a control experiment. This is because the reinforcing coiled fibres in Composite 3 are of the highest crystallinity and able to produce the highest blocking force [24]. Controlled and scalable damping behaviour can be obtained by changing the geometrical, physical, and/or mechanical conditions of the coiled fibres, such as coil angle, fibre diameter, annealing temperature, and pre-stretching the coils.

## 4. Conclusions

In this paper, a self-reinforced nylon 6 composite has been developed and its vibration dampening behaviour was investigated. The reinforcing-oriented nylon 6 fibres were in coiled shape with a thermoresponsive actuating property. A continuous phase of unoriented nylon 6 matrix was 3D printed impregnating the coiled fibres. The slightly higher melting point of coiled fibres (222 °C) ensured that there was no property damage of these fibres while 3D printing the matrix nylon 6 with a melting point of 218 °C. The blocking force generated within the reinforcing coiled actuator is responsible for dissipating vibration energy and increasing the magnitude of the damping factor compared to a non-reinforced nylon 6. The most promising result was found when the reinforcing coils were heat set at 150 °C before composite fabrication. These fibres could produce a higher blocking force due to their enhanced polymer crystallinity and damping properties, thereby showing higher vibration dampening capability. The coiled actuator provides an enhanced dampening capability to the composites. The extent of crystallinity of the reinforcing actuator is found to directly influence the vibration dampening capacity.

The current study mostly focused on the development of new smart composite material for adaptive vibration damping in a heated system and further investigations and improvements of the composite systems are necessary to suit them to real-world applications. Our future investigations will be on maximising the vibration damping capacity by altering the geometry of the coiled fibres as this directly influences the capacity of producing blocking force. Another approach to maximise the damping performance will also be studied by fabricating self-reinforced composites with different thermoplastic polymers and changing the infill geometries during the 3D printing of matrix material. It is also well-established that the vibration damping performance has a dependency on the system temperature, and we anticipate conducting such investigations in our extended works on the currently developed novel vibration damper. 

## Figures and Tables

**Table 1:** DSC analysis results of nylon 6 fibres.

Sample	Melting Temperature (°C)	Area of Melting Peak (J/g)	Degree of Crystallinity (%)
As-received	225.3(±0.08)	85.23 (±0.21)	35.7 (±0.05)
Coiled: Heat set @ 50 °C	221.96 (±0.06)	77.32 (±0.18)	32.3 (±0.1)
Coiled: Heat set @ 100 °C	221.87 (±0.08)	78.63 (±0.22)	32.9 (±0.09)
Coiled: Heat set @ 150 °C	223.47 (±0.1)	82.43 (±0.27)	34.5 (±0.1)
Coiled: Heat set @ 200 °C	222.31 (±0.12)	80.83 (±0.23)	33.8 (±0.11)

## Data Availability

The data presented in this study are available on request from the corresponding authors.
